# Prevalence and Predictors of Asymptomatic Malaria Parasitemia among Pregnant Women in the Rural Surroundings of Arbaminch Town, South Ethiopia

**DOI:** 10.1371/journal.pone.0123630

**Published:** 2015-04-07

**Authors:** Desalegn Nega, Daniel Dana, Tamirat Tefera, Teferi Eshetu

**Affiliations:** 1 Malaria and Other Parasitic and Vector-Borne Diseases Research Team, Directorate Of Parasitic, Bacterial and Zoonotic Diseases Research, Ethiopian Public Health Institute, Addis Ababa, Ethiopia; 2 Department of Medical Laboratory Sciences and Pathology, College of Public Health and Medical Sciences, Jimma University, Jimma, Oromia, Ethiopia; Institut de Recherche pour le Développement, FRANCE

## Abstract

**Background:**

In Sub-Saharan African countries, including Ethiopia, malaria in pregnancy is a major public health threat which results in significant morbidities and mortalities among pregnant women and their fetuses. In malaria endemic areas, *Plasmodium* infections tend to remain asymptomatic yet causing significant problems like maternal anemia, low birth weight, premature births, and still birth. This study was conducted to determine the prevalence and predictors of asymptomatic *Plasmodium* infection among pregnant women in the rural surroundings of Arba Minch Town, Southern Ethiopia.

**Methods:**

A community based cross-sectional study comprising multistage sampling was conducted between April and June, 2013. Socio-demographic data were collected by using a semi-structured questionnaire. *Plasmodium* infection was diagnosed by using Giemsa-stained blood smear microscopy and a rapid diagnostic test (SD BIOLINE Malaria Ag Pf/Pv POCT, standard diagnostics, inc., Korea).

**Results:**

Of the total 341 pregnant women participated in this study, 9.1% (31/341) and 9.7% (33/341) were confirmed to be infected with *Plasmodium* species by microscopy and rapid diagnostic tests (RDTs), respectively. The geometric mean of parasite density was 2392 parasites per microliter (*μl*); 2275/ μl for *P*. *falciparum* and 2032/ μl for *P*. *vivax*. Parasitemia was more likely to occur in primigravidae (Adjusted odds ratio (AOR): 9.4, 95% confidence interval (CI): 4.3–60.5), secundigravidae (AOR: 6.3, 95% CI: 2.9–27.3), using insecticide treated bed net (ITN) sometimes (AOR: 3.2, 95% CI: 1.8- 57.9), not using ITN at all (AOR: 4.6, 95% CI: 1.4–14.4) compared to multigravidae and using ITN always, respectively.

**Conclusion:**

Asymptomatic malaria in this study is low compared to other studies’ findings. Nevertheless, given the high risk of malaria during pregnancy, pregnant women essentially be screened for asymptomatic *Plasmodium* infection and be treated promptly via the antenatal care (ANC) services.

## Background

Malaria is the most deadly tropical infectious disease disproportionately affecting the poor, children under the age of 5 years, and pregnant women. There were an estimated 216 million episodes of malaria worldwide in 2010, of which approximately 81% or 174 million cases were in the African region [1].

In malaria endemic areas, a significant proportion of individuals have asymptomatic infection with *Plasmodium* species [2, 3] among whom pregnant women are at higher risk [4]. The sequestration of *Plasmodium* species in placenta is believed to be associated with low birth weight, preterm delivery, miscarriage, and still birth [5, 6]. Besides, asymptomatic carriers serve as silent reservoir of gametocytes for transmission by mosquito vectors [7, 8].

Malaria during pregnancy remains a major public health threat in sub-Saharan Africa where about 125 million pregnancies are at risk of malaria each year, and up to 200,000 babies die as a result [9].

Use of long-lasting insecticidal nets (LLINs), the administration of intermittent preventive treatment with sulfadoxine-pyrimethamine (IPTp-SP), and appropriate case management through rapid and effective therapy of malaria in pregnant women are the current strategies recommended by The World Health Organization (WHO) [10]. Currently, WHO strives to increase access to IPTp-SP for pregnant women in all areas with moderate to high malaria transmission in Africa, as part of ANC service package [11].

In Ethiopia, an estimated 55.7 million people (68% of the population) are at risk of malaria, and three fourth of the land mass is considered malarious [12]. The Federal Ministry of Health (FMOH) estimates annual cases of clinical malaria as 5–10 million accounting for 12% of outpatient consultations and 10% of hospital admissions [13]. In most of the areas malaria transmission is unstable leading to epidemics. In the country *Plasmodium falciparum* and *Plasmodium vivax* are the main species accounting for roughly 60 and 40% of malaria cases, respectively [14] though recent reports indicate the shift of dominance from *falciparum* to *vivax* in highland areas [15, 16].

In Ethiopia, early diagnosis and prompt treatment is one of the key strategies in controlling malaria. Blood smear microscopy and malaria rapid diagnostic tests (RDT) represent the two diagnostics most widely used. RDT is used at health post level in rural areas where microscopy cannot be used. The mass distribution of insecticide-treated bed nets (ITNs), together with increased utilization of long-lasting ITNs (LLINs), indoor residual spraying (IRS) and adoption of Artemisinin-based combination therapy (ACT) resulted in substantial declines in malaria-related deaths in Ethiopia [17].

To the best of our knowledge there has been no published study to attest the epidemiological data regarding asymptomatic malaria parasitemia among pregnant women in the study area. Thus, the present study determined the prevalence of asymptomatic malaria parasitemia and its predictors among pregnant women in the rural District surrounding Arba Minch Town, Southern Ethiopia.

## Materials and Methods

### Study area

This study was conducted at the rural district surrounding Arbaminch Town, which is one of the Districts in the Southern Nations, Nationalities, and Peoples' Region of Ethiopia. The Arba Minch town lies in tropical climatic zone with an altitude of 1,200–1,300 meter ASL, an average annual temperature of 29.7°C and rainfall of 700 mm. The District consists of 30 villages, of which 11 have been known malarious villages with intense transmission pattern [18].

### Study design, inclusion and exclusion criteria

A community based cross-sectional study was conducted among apparently healthy pregnant women. Pregnant women with absence of disease symptom/sign within the past 48 hours, axillary temperature ≤ 37.5°c, permanent residents in the study area, and those willing to participate in the study and signed the informed consent were included. Individuals having taken anti-malarial drugs in the past six weeks prior to data collection, those undergoing any kind of long term medical treatments, and unwilling individuals were excluded from the study.

### Sample size determination and sampling technique

The required sample size for this study was calculated using a formula for a single population proportion. Taking expected malaria prevalence of 11.59% [19], 95% confidence level and 5% margin of error, the calculated sample size was 158. By taking a design effect of 2 and non-response rate of 10%, the total sample size became 346.

**Fig 1:**
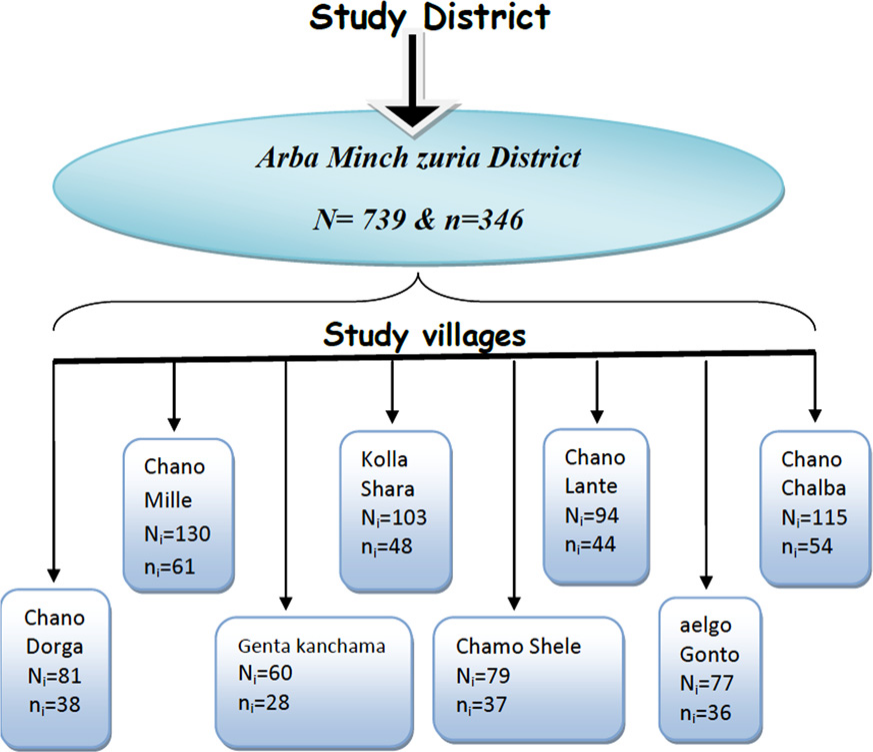
Flow chart indicating the sampling procedure for a study conducted on asymptomatic malaria parasitemia among pregnant women in rural surroundings of Arba Minch, Southern Ethiopia, between April and June 2013.

Multistage sampling of the study subjects in eight Study villages, from the known 11 malarious villages in the District, were selected by simple random sampling using the lottery method. The sample size was then distributed proportionally to the villages based on the size of their pregnant women population (Fig 1). The selected study villages were: Chano Mile, Chano Dorga, Chamo Shele, Chano Chalba, Aelgo Gonto, Genta Kenchama, Chano Lante, and Kolla Shara.

At the village level, the households were selected by simple random sampling by using the sampling frame which was prepared after having identified the pregnant women in the households by the preliminary assessment through active house-to-house visits. Computer generated random numbers were used for the random selection of the study households or study participants. In the cases where more than one eligible woman was encountered in a single household, a lottery method was used to select a woman to be recruited to the study. The health extension workers guided us in the preliminary assessment by indicating the houses where pregnant women were living.

### Data collection

A pre-tested semi-structured questionnaire was administered by trained interviewer to obtain data on socio demographic characteristics of the pregnant women. Capillary blood samples were collected by a finger pricking using disposable lancet.

### Laboratory investigations

Giemsa stained blood smear microscopy and RDTs were employed for the diagnosis of asymptomatic malaria parasitemia. Parasite density per microliter (μl) was determined by counting the number of parasites per 200 white blood cells on a thick blood film assuming a total standard WBC count of 8000/μl. The degree of parasite density was graded as mild, moderate, and severe when the counts were between 1–999 parasites/*μl*, 1000–9999/μl, and >10,000/μl, respectively, following the method described elsewhere [20].

$$Parasites/\mu l\;=\;\frac{No.\;of\;asexual\;stages\;\times \;8000\;leukocytes}{200\;leukocytes}$$

The rapid diagnostic test used in this study was SD BIOLINE Malaria Ag P.f/P.v POCT standard diagnostics, Inc., Korea. This test is one step, rapid, qualitative, and differential test for the detection of HRP-II (Histidine-rich protein II) specific to *P*. *falciparum*, and *p*LDH (*Plasmodium* lactate dehydrogenase) specific to *P*. *vivax* in a human blood sample.

### Ethical considerations

The study protocol was approved by the ethical review board of Jimma University, Ethiopia. Community agreement and local oral consent was obtained from community leaders. Written informed consent was obtained from all of the study participants. For individuals under the age of 18, written informed consent was obtained from their husbands. Data collected from each study participant and results of laboratory tests were kept confidential and used only for the research purpose. Result of participants with parasitic infection was addressed to the study participants. The pregnant women who were found to be infected with *Plasmodium* parasite were referred for treatment and medical consultation in the ANCs of nearby health facilities and followed up to ensure appropriate treatment.

### Statistical analysis

Data were coded, entered into, cleaned and analyzed using SPSS for windows version 16.0. Both descriptive and inferential statistics were employed for the analysis of data. Frequencies were used to determine the prevalence of asymptomatic *Plasmodium* infection in pregnant women. Binary logistic regression was employed to assess the predictors of asymptomatic *Plasmodium* infection. Variables significant at P-value of 0.25 in the univariate logistic regression were selected for Multivariate logistic regression analysis model. Odds ratios with 95% confidence intervals were calculated and P- value < 0.05 was considered to be statistically significant. Cohen’s kappa coefficient (k) was used to determine the degree of agreement between the diagnostic methods. Shapiro-Wilk test of normality was applied for continuous variables.

### Quality control

Two experienced laboratory technologists individually examined the microscopic slides. Hundred microscopic fields of the thick smear were examined before concluding as negative. Discrepancy between the first and second readings was settled by a third senior microscopist, whose readings were considered final. The manufacturer’s instruction was strictly followed for the RDTs. Blood smear microscopy readers were blinded to the result of RDTs.

## Results

### Socio demographic characteristics of the pregnant women

A total of 341 pregnant women were involved in this study giving a response rate of 98.6%. The age of the participants ranged from 17 to 40 years with a median age of 25 (Inter-quartile range: 23–29), majority (41.1%) of them between 21–25 years. Majorities (95%) of the women were married and about 70% of the women completed their primary and secondary school education. The mean family size in this study was 4.5 (SD ±1.9). Twenty six percent of the subjects were primigravidae, 34.6% of the pregnant women were in the third trimester and more than 80% of the subjects attended ANC (Table 1).

**Table 1:** Socio-demographic characteristics of the pregnant women in the rural surroundings of Arbaminch Town, south Ethiopia between April and June 2013.

Variables	No. (%)	Variables	No (%)
**Age groups**		**Marital status**	
≤20	32(9.4)	Married	327(95.9)
21–25	140(41.1)	Single	4(1.2)
26–30	102(29.9)	Divorced	6(1.8)
31–35	47(13.8)	Widowed	4(1.2)
>35	20(5.9)	**Gestational age**	
**Parity**		1^st^ trimester	83(24.3)
Primigravidae	91(26.7)	2^nd^ trimester	140(41.1)
Secundgravidae	99(29.0)	3^rd^ trimester	118(34.6)
Multigravidae	151(44.3)	**Education**	
**Occupation**		Illiterate	59(17.3)
Farmer	98(28.7)	Read/write	15(4.4)
Daily laborer	40(11.7)	Primary	134(39.3)
Merchant	57(16.7)	Secondary	95(27.9)
House wife	134(39.3)	College/above	38(11.1)
Civil servant	12(3.5)	**ITN use**	
**Family size**		Use always	187(54.8)
1–3	116(34.0)	Use rarely	19(5.6)
4–7	168(49.3)	Do not use	135(39.6)
>7	57(16.7)	**IRS(past 12 months)**	
**ANC Attendance**		Yes	289(84.8)
Yes	275(80.6)	No	52(15.2)
No	66(19.4)		

### Parasitemia among asymptomatic pregnant women

The prevalence of asymptomatic plasmodium infection was determined as 9.1% (31/ 341) and 9.7% (33/341) by using microscopy and RDTs, respectively. The species identified from thin blood smear include: 12(38.71%) *P*. *falciparum*, 15(48.38%) *P*.*vivax*, and 4(12.9%) mixed infections. Mixed infection was observed among primigravidae, secundigravidae, and women in their 2^nd^ trimester (Table 2). The density of parasitemia, as determined from the thick blood smear, ranged from 720 to 10120 parasites /μl. The geometric mean of parasite density among the 31 parasitemic pregnant women was 2392/*μl* (95% CI: 1790–3165); 2275/ μl for *P*. *falciparum*, and 2032/ μl for *P*. *vivax*. Of the 31 diagnosed of asymptomatic *Plasmodium* infection; 5(16.1%), 23(74.2%), and 3(9.7%) had mild, moderate, and severe parasitemia, respectively. Severe parasitemia occurred among primigravidae and individuals in their 1^st^ and 2^nd^ trimester (Table 3). The species diagnosed as positive by RDTs were 13 (39.4%) *P*. *falciparum*, 16 (48.5%) *P*. *vivax*, and 4(12.1%) mixed infections.

**Table 2:** Distribution of *Plasmodium* species with regard to trimester and gravidity among pregnant women in the rural surroundings of Arbaminch Town, South Ethiopia between April and June 2013.

Characteristics		Parasite species				p-value
		*P*.*falciparum*	*P*.*vivax*	Mixed	Total (%)	
Trimester	1^st^	3	3	0	6 (19.4)	0.676
	2^nd^	6	6	3	15 (48.4)	
	3^rd^	3	6	1	10 (32.2)	
Gravidity	primigravidae	7	5	2	14 (45.2)	0.456
	secundigravidae	2	5	2	9 (29.0)	
	multigravida	3	5	0	8(25.8)	

**Table 3:** Degree of parasitemia with regard to Trimester and gravidity among pregnant women in the rural surroundings of Arbaminch Town, South Ethiopia between April and June 2013.

Characteristics		Parasitemia				p-value
		Mild	moderate	Severe	Arithmetic mean /Ul	
Trimester	1^st^	2	3	1	3560	0.449
	2^nd^	2	11	2	3400	
	3^rd^	1	9	0	2684	
Gravidity	Primigravidae	2	9	3	4062	0.325
	secundigravidae	1	8	0	2680	
	Multigravidae	2	6	0	2275	

### Diagnostic performance of RDTs against light microscopy

The diagnostic performance of SD BIOLINE Malaria Ag *P*.*f/P*.*v* POCT was assessed by taking blood smear microscopy as a reference method. The sensitivity, specificity, positive predictive value, negative predictive value, and efficiency of the RDTs used in the present study were 100%, 99.3%, 93.9%, 100%, and 99.4%, respectively. A good measure of agreement (k = 0.96) was observed between the SD BIOLINE Malaria Ag *P*.*f/P*.*v* POCT and light microscopy.

### Univariate and multivariate logistic regression analysis of predictors

Socio-demographic characteristics and other health related factors were analyzed using binary logistic regression for possible association and presented in Table 4. Gravidity, ITN usage, and age group showed significant association with *Plasmodium* infection at P-value of < 0.05.

**Table 4:** Multivariate logistic regression analysis of predictors for asymptomatic *Plasmodium* infection among pregnant woman in rural surroundings of Arba Minch, Southern Ethiopia, between April and June 2013.

Variables	No. examined (%)	Pos (%)	COR (95%CI)	P-value	AOR (95%CI)	p-value
**Age groups**						
**<20**	20 (5.9)	2(10)	0.44 (0.07, 2.76)	0.38	0.08 (0.05, 3.45)	0.423
**20–35**	301(88.3)	25(8.3)	0.36 (0.11, 1.16)	0.08	0.17 (0.01, 1.56)	0.111
**35+**	20(5.9)	4(20)	1		1	
**Occupation**						
**Farmer**	98(28.7)	9(9.2)	0.30(0.07–1.33)	0.113*	0.55(0.06–4.75)	0.586
**Daily laborer**	40(11.7)	10(25.0)	1.00(0.23–4.44)	1.000	2.94(0.29–29.67)	0.360
**Merchant**	57(16.7)	3(5.3)	0.17(0.03–0.96)	0.045*	0.69(0.07–7.11)	0.756
**House wife**	134(39.3)	6(4.5)	0.14(0.03–0.66)	0.013*	0.29(0.04–2.22)	0.231
**Civil servant**	12(3.5)	3(25.0)	1.00		1.00	
**Education**						
**Illiterate**	59(17.3)	10(16.9)	1.35(0.42–4.30)	0.615	0.47(0.06–3.59)	0.463
**Read/write**	15(4.4)	3(20.0)	1.65(0.34–7.98)	0.534	0.76(0.06–9.17)	0.829
**Primary**	134(39.3)	9(6.7)	0.48(0.15–1.51)	0.208*	0.44(0.08–2.51)	0.351
**Secondary**	95(27.9)	4(4.2)	0.29(0.07–1.15)	0.077*	0.23(0.03–1.62)	0.142
**College/above**	38(11.1)	5(13.2)	1.00		1.00	
**Family size**						
**1–3**	116(34.0)	14(12.1)	1.00		1.00	
**4–7**	168(49.3)	11(6.5)	0.51(0.22–1.17)	0.111*	1.92(0.40–9.26)	0.419
**>7**	57(16.7)	6(10.5)	0.86(0.31–2.36)	0.766	2.16(0.31–14.88)	0.435
**Parity**						
**Primigravidae**	91(26.7)	14(15.4)	3.25(1.30–8.09)	0.011*	9.40(4.30–60.53)	0.000**
**Secondgravida**	99(29.0)	9(9.1)	1.79(0.67–4.80)	0.249*	6.34(2.98–27.30)	0.001**
**Multigravida**	151(44.3)	8(5.3)	1.00		1.00	
**ITN use**						
**Use always**	187(54.8)	6(3.2)	1.00		1.00	
**Use sometimes**	19(5.6)	5(26.3)	10.77(2.92–39.75)	0.000*	3.22(1.80–57.95)	0.009*
**Do not use**	135(39.6)	20(14.8)	5.25(2.05–13.46)	0.001*	4.61(1.48–14.41)	0.009*
**IRS(past 12 months)**						
**Yes**	289(84.8)	22(7.6)	1.00		1.00	
**No**	52(15.2)	9(17.3)	2.54(1.10–5.88)	0.03*	2.19(0.62–7.76)	0.225

* Significant at p-value < 0.25

** Significant at p-value < 0.05

After adjusting for possible confounders; the odds of being infected with *Plasmodium* was 9.4 times (95%CI: 4.3–60.5) higher among primigravidae, 6.3 times (95% CI: 2.9–27.3) higher among secundigravidae as compared to multigravidae (Table 4). There was no significant association between gestational age, age group, and asymptomatic *Plasmodium* infection among the pregnant women.

ITN usage was another factor assessed for possible association with asymptomatic *Plasmodium* infection among the pregnant women. It was found that the odds of *Plasmodium* infection was 3.2 times (95% CI: 1.8–57.9) higher among women who were using ITN sometimes and 4.6 times (95% CI: 1.4–14.4) higher among those who did not use ITN at all as compared to those who were using ITN always (Table 4).

The univariate analysis showed that IRS was inversely associated with prevalence of asymptomatic *Plasmodium* infection (OR: 2.5, 95% CI: 1.1–5.8). Yet it was unable to independently predict the risk of asymptomatic *Plasmodium* infection after adjusting for others confounders (OR: 2.1, 95% CI: 0.6–7.7) (Table 4).

Parasitemia was more common in the 2^nd^ trimester where 10.7% (15/140) were positive, followed by those in 3^rd^ trimester with 8.5% (10/118) positive, and the lowest rate was in the 1^st^ trimester with 7.2% (6/83) were found to be positive. However, these differences were not statistically significant (*X* ^2^ = 0.849, p = 0.654).

## Discussion

The present study aimed at assessing asymptomatic *Plasmodium* infection among pregnant women in the rural District surrounding Arbaminch town, Southern Ethiopia. The study revealed prevalence of asymptomatic infection with *P*. *falciparum or P*. *vivax* among the women as 9.1% and 9.7% by using Giemsa stained blood smear microscopy and the SD BIOLINE Malaria Ag *P*.*f/P*.*v* POCT test, respectively. This prevalence is in agreement with the report of malaria prevalence among pregnant women ranged from 10% to 65% in malaria endemic areas [21].

The finding of the present study corroborates the findings of similar studies reported from other parts of Ethiopia [22, 23]. However it is lower than the finding of 27% by RDTs and 23% by microscopy reported from Democratic Republic of the Congo [4], and is higher than the prevalence of 3.1% by microscopy and 4.8% by RDTs reported from Nigeria [24]. This might be due to differences in geographical locations or transmission pattern. In different geographical locations, there are unique malaria transmission patterns that result in different immune acquisition capacity of the residents. Individuals living in higher malaria transmission areas have greater chance of developing asymptomatic malaria because they get frequent infections that can highly boost immunity against malaria, while those in low transmission areas have low infection frequency thus there may occur low prevalence of asymptomatic malaria [25].

In the present study, a good measure of agreement between microscopy and RDTs was obtained. The SD BIOLINE Malaria Ag P.f/P.v POCT test had a sensitivity of 100% and a specificity of 99.35% for the detection of asymptomatic malaria compared to microscopy. This study results agree with findings from similar studies conducted elsewhere [26, 27].

The ownership of ITN in the present study is 60.4%, though usage pattern differs among the study subjects. This is lower than the report of 89.6% by Getachew and his colleagues [22].

There was a strong association between increasing gravidity and decreasing rates of parasitemia. This agrees with findings of similar studies from sub-Saharan African countries where the prevalence of asymptomatic *Plasmodium* infection was significantly higher in primigravidae than the multigravidae [28, 29]. It might be linked to infection-specific immunological factors. Some *Plasmodium* infected erythrocytes sequester in the maternal placenta by producing surface antigens, mainly variant surface antigen, that adhere to chondroitin sulphate-A (CSA) receptors expressed by syncytiotrophoblasts in the placenta. Primigravidae and secundigravidae are more susceptible to infection, as they lack these anti-adhesion antibodies against CSA-binding parasites, which develop only after successive pregnancies [30].

In the present study there was no significant difference in the rate of asymptomatic malaria parasitemia with respect to age of the pregnant women. However, the findings of some similar studies [17, 31] suggested that peripheral parasitemia was higher in pregnant women of younger age groups than old ages. This contrast might be due to differences in the sample size, sampling technique, physiologic and biochemical factors of pregnant women and the study setting such as geographical differences, altitude,temperature, and age categorization scheme.

The fact that this study is community based and built-in multistage probability sampling technique increases the chance of generalization to the whole pregnant population in the district. Besides, incorporation of the two diagnostic methods in detection of asymptomatic malaria among the pregnant women is also one of the strong side of this study. However, the use of more sensitive and specific technique such as nested PCR is recommended for detection and identification of asymptomatic *Plasmodium* infection [32].

Malaria has a low to moderate transmission pattern in Ethiopia. In countries where malaria has moderate to high transmission, WHO recommends intermittent preventive treatment with sulfadoxine pyrimethamine (IPTp-SP) for pregnant women as part of the ANC services, which does not apply for Ethiopia[11]. Hence, screening of pregnant women for asymptomatic *Plasmodium* infection should be the way forward to fight against malaria in pregnancy and its consequences on fetus.

The present study has its own limitations; the study period was during the minor malaria transmission season. The wider confidence interval in the calculation of odds ratio might indicate the inadequacy of the sample size. Besides, due to the study design (a cross-sectional study) it could not underscore the health impact of asymptomatic *Plasmodium* infection among the pregnant women. Hence a longitudinal study covering both the major and minor malaria transmission season and with an objective of assessing the outcome of asymptomatic *Plasmodium* infection is needed.

## Conclusion

The findings of the present study suggests the need of further malaria control strategies to screen the pregnant women for asymptomatic *Plasmodium* infection followed by prompt malaria treatment in the ANC service package. Such initiatives not only safeguard the pregnant women from malaria associated morbidities and mortalities but also the fetus and the newborns. As part of ANC service package, educating on the appropriate usage and benefits of the bed nets, and encouraging early ANC attendance among pregnant women could enhance benefits for the women’s health. RDTs can be used sufficiently for diagnosis of the asymptomatic malaria in areas where there is no access to light microscopy.
